# MMP-9, a Potential Target for Cerebral Ischemic Treatment

**DOI:** 10.2174/157015909790031157

**Published:** 2009-12

**Authors:** Xue Dong, Yu-Ning Song, Wei-Guo Liu, Xiu-Li Guo

**Affiliations:** 1 Department of Pharmacology, School of Pharmaceutical Sciences, Shandong University, No. 44 WenHuaXi Road, Jinan 250012, P.R. China; 2 Qianfoshan Hospital, Jinan 250014, P.R. China

**Keywords:** Matrix metalloproteinase-9 (MMP-9), cerebral ischemia, regulation, therapeutical target.

## Abstract

Matrix metalloproteinase-9 (MMP-9) which is a member of matrix metalloproteinases family that normally remodel the extracellular matrix, has been shown to play an important role in both animal models of cerebral ischemia and human stroke. The expression of MMP-9 is elevated after cerebral ischemia which is involved in accelerating matrix degradation, disrupting the blood-brain barrier, increasing the infarct size and relating to hemorrhagic transformation. Recently, many drugs, such as tetracycline derivatives, cyclooxygenase inhibitors, ACEI inhibitors and AT1 receptor blockers, etc., have been found to attenuate the elevated expression levels of MMP-9 after ischemia and to reduce the damage of cerebral ischemic. This article reviews the physiological features of MMP-9 and its important role in the genesis, propagation, and therapeutics of cerebral ischemic diseases.

## 1. INTRODUCTION

Matrix metalloproteinases (MMPs) are a family of zinc- and calcium-dependent proteolytic enzymes that normally remodel the extracellular matrix. MMPs cleave most components of the extracellular matrix including fibronection, laminin, proteoglycans and type IV collagen [56]. An overexpression of MMPs followed by accelerated matrix degradation is associated with several pathologies including cancer cell invasion and metastasis, the loss of cartilage in osteoarthritis, rheumatoid arthritis, cardiovascular diseases, acute lung injury, chronic obstructive pulmonary disease, eye and skin diseases and periodontitis [15].

Among MMPs, matrix metalloproteinase-9 (MMP-9) /gelatinase B has been proved to play an important role in wound healing, angiogenesis, inflammation, tumor invasion and metastasis [51]. And during the last decade, an abnormal expression of MMP-9 has been shown to play a deleterious role in brain injury in both animal models of cerebral ischemia and human stroke. Furthermore, MMP-9 knockout models or treatment with MMP tissue inhibitors, synthetic MMP inhibitors and MMP neutralizing antibodies have been shown to protect blood-brain barrier (BBB), reduce vaso-genic edema formation and infarct size after cerebral ischemia [1, 2, 38, 49, 52, 58, 71]. These suggest that MMP-9 might be an important clinical target for the therapy of human cerebral ischemia. Furthermore, several experiments have been carried out to investigate the relationship between some medicines and MMP-9 expression levels. Researchers expect to find some new drugs for the treatment of ischemic diseases.

## 2. THE MOLECULAR STRUCTURE AND PHYSIOLOGICAL FUNCTION OF MMP-9

MMPs are a group of homogeneous enzymes that degrade molecules of the extracellular matrix (ECM). They are grouped into collagenases, gelatinases, stromelysins, matrilysins, membrane type (MT)-MMPs and others basing on domain organization and substrate preference [45]. Gelatinase A (MMP-2) and gelatinase B (MMP-9) belong to the gelatinases group. The MMPs contain several distinct domains that are responsible for secretion, latency, catalysis and substrate recognition. All MMPs share one pre-domain and one catalytic domain. The pre-domain is required to maintain latency of these enzymes and is cleaved off upon activation. The following catalytic domain contains the zinc-binding motif, with three conserved histidine residues which complex the critical metal ion and the catalytic domain contains additional zinc and calcium ions which maintain the three dimensional structure of MMPs and are necessary for stability and enzymatic activities [9]. The gelatinases group contains an additional three repetitional fibronectin type II motifs inserted into the catalytic domain which suggests to facilitate the binding of these enzymes to their substrates gelatin and collagen [45]. In MMP-9, a unique linker sequence, which is more than 50 amino acids long, connects the active site and the hemopexin domain. It has been considered to be an independent protein domain that has low homology with type V collagen. The linkage domain is required to correctly orient the hemopexin domain for inhibition by TIMP-1 and internalization by LRP-1 and megalin. Therefore, the linkage and hemopexin domains down-regulate the bioavailability of active MMP-9. And interactions with the cargo receptors are proposed to be the original function of hemopexin domains [66]. The domain structure of MMP-9 is illustrated in Fig. (**1A**). MMP-9 are initially synthesized and secreted as inactive zymogen pro-MMP-9 and activated by cleaving the prodomain so that most experiments about MMP-9 can detect two forms of MMP-9: one at about 92kDa, which corresponded to pro-MMP-9, another at 88kDa, corresponding to an activated MMP-9. MMP-9 has proteolytic activity and degrade denatured collagens, gelatins and a number of ECM molecules including type IV, V and XI collagens, laminin and aggrecan core protein [45].

## 3. MMP-9 AND CEREBRAL ISCHEMIA

The expressions of MMP-9 and MMP-2 are elevated after cerebral ischemia and able to open the BBB [50, 52]. In rodent models, MMP-9 responses appear to dominate in the acute phase, whereas MMP-2 elevations seem to occur in the delayed phase, days after stroke [50]. Moreover, recent data confirm the presence of high MMP-9 levels not only in infarct tissue but also in the peri-infarct areas, suggesting that MMP-9 involve in the process of infarct growth [55]. Furthermore, MMP-9 level seems to peak within infarcts that undergo hemorrhagic conversion, correlating with enhanced erythrocyte extravasation and neutrophil infiltration surrounding the affected capillaries together with severe collagen IV degradation in the basal lamina [53]. Although in the brain, gelatinases have been the most intensively studied because of the ease with which they can be identified by gelatin zymography and their prominent role in injury and repair [57], other MMP members may play important roles as well [54]. For example, MMP-3 can be activated after ischemia-reperfusion in rat brain, causing the cleavage of the cerebral matrix agrin and contribute to BBB opening during neuroinflammation after intracerebral lipopolysaccharide (LPS) injection in mice [23, 60]. Whatever, in this article we just focus on MMP-9 in cerebral ischemia.

### 3.1. MMP-9 and Blood-Brain Barrier Opening

Blood-brain barrier (BBB) plays an important role in protecting the neuronal microenvironment. Endothelial cells of brain capillaries have tight junctions, which can restrict molecules from moving between the blood and brain. The extracellular matrix molecules constitute the basal lamina which is around the capillary with pericytes. MMP-9 can degrade a number of extracellular matrix molecules to breakdown the BBB. When the integrity of BBB is lost, inflammatory cells and fluid penetrate the brain, causing hemorrhage, vesogenic edema and neuronal cell death.

A lot of studies have shown that the expression of MMP-9 increases after permanent middle cerebral artery occlusion (pMCAO) and transient middle cerebral artery occlusion (tMCAO). Significant MMP-9 activity was observed at 12 hours and reached maximum levels by 24 hours, then persisted for 5 days at this level and returned to basal (zero) levels by 15 days using a model of pMCAO in rats [52]. Also a finding demonstrated pro-MMP-9 expression was significantly increased in ischemic regions compared with corresponding contralateral regions after 2 hours of ischemia and remained elevated until 24 hours and activated MMP-9 was observed 4 hours after ischemia. Moreover, at the same time as the appearance of activated MMP-9, a clear increase of BBB permeability was detected. This suggested that MMP-9 may play an active role in early vasogenic edema development after stroke [18]. The study with 50 minutes tMCAO rats showed an increase of MMP-9 from 4 hours to 4 days after reperfusion [50]. In rats with 2 hours tMCAO, a maximally increase in MMP-9 was associated with maximal brain sucrose uptake at 48 hours of reperfusion [58]. There is the discrepancy among these studies and this can be explained by technical differences or different animals and different testing time phase. Recently, a research showed a strong neutrophil infiltration in the infarcted and hemorrhagic areas with local high MMP-9 content closely related to basal lamina collagen IV degradation and blood-brain barrier breakdown [54].

### 3.2. MMP-9 and Cerebral Infarction

Brain injury after middle cerebral artery occlusion (MCAO) is primarily a result of the decrease in blood flow and energy depletion. Then the loss of oxygen and glucose result in the neuronal cell death within minutes in the core zone, an area of severe ischemia. The ischemic penumbra, the hypoperfusion region associated with the focal ischemia, is variable. This area can be recovery or become infarction after reperfusion. 

Recently, some studies showed that the activation of MMP-9 relate to the infarct size. Pro-MMP-9 levels in plasma and activated MMP-9 levels in brain homogenates were progressively increased over the course of 24 hours after permanent middle cerebral artery occlusion in male Sprague-Dawley rats. And plasma levels of pro-MMP-9 at 24 hours were correlated with final infarct volumes [48]. A decrease in infarct size after focal ischemic insult in rats was observed when a MMP-9 neutralizing monoclonal antibody was administered [52]. Serial MMP-9 and MMP-2 in 39 patients with cardioembolic stroke were determined. Results suggested that higher MMP-9 levels were associated with neurological deterioration during the first 48 hours and a positive correlation was between mean MMP-9 and total infarct volume [40]. Several inflammatory families such as proinflammatory cytokines (TNF-α, IL-6), adhesion molecules (ICAM-1), chemokines (IL-8), or matrix metalloproteinases (MMP-2, MMP-9) of 16 patients with acute middle cerebral artery stroke were studied. Among them, MMP-9 was found to be the most powerful and only predictor of the infarct volume measured as a diffusion-weighted magnetic resonance imaging lesion [43]. The serum levels of MMP-2, MMP-3, MMP-9, MMP-13, TIMP-1, TIMP-2 and laminin in 50 patients with acute ischemic stroke were measured and results demonstrated that levels of MMP-9 and laminin varied significantly by infarct size [26]. These studies certified the increased of MMP-9 in infracted tissue both in animal experiments and human stroke. Thus, MMP-9 can be a potential therapeutic target for the treatment of stroke.

### 3.3. MMP-9 and Hemorrhagic Transformation

Plasminogen activators, including tissue plasminogen activator (tPA) and urokinase plasminogen activator (uPA), are serine proteases that activate plasminogen into plasmin. Recombinant tPA (rtPA) is beneficial in ischemic stroke when thrombolytic therapy is started within 3 hours after symptom onset. On the other hand, thrombolysis is associated with the risk of hemorrhage transformation. 

A lot of studies about the relationships between tPA and MMPs show that the plasminogen-plasmin system might be involved in activation of MMPs. Administration of heparin for 3 hours after MCAO increased tPA and MMP-9 activity and their mRNA expression in wild-type mice but not in tPA deficient knockout mice [72]. Closure of the BBB with broad-spectrum MMP inhibitor BB-94 given before rtPA treatment reduced rtPA-mediated mortality in 6/12 to 33% [49]. Recently, a study demonstrated for the first time that the injection of a MMP inhibitor for 3 hours after the ischemia in rats significantly decreased the brain edema and reduced the risk of hemorrhagic transformation after thrombolysis with rtPA [11]. A study published in 2001 firstly showed an association between MMP-9 expression and several subtypes of hemorrhagic transformation after human cardioembolic stroke [41]. Subsequently, a study demonstrated that high plasma levels of MMP-9 are independently associated with hemorrhagic transformation in acute ischemic stroke by studying 38 patients [8]. Moreover, the baseline MMP-9 level was the only factor independently associated with late hemorrhagic infarction among patients they observed [41]. Therefore, the baseline MMP-9 level predicts parenchymal hemorrhagic appearance after t-PA treatment [42]. The findings above suggest that the endogenous tPA or rtPA treatment, through the enhancement of MMP-9 expression, play an important role in hemorrhagic transformation after cerebral ischemia. MMP inhibitors can be used before thrombolytic therapy to reduce hemorrhagic transformation.

### 3.4. MMP-9 and Stroke Recovery

MMP-9 promotes injury of the BBB, vasogenic edema formation, infarct size and hemorrhagic transformation in the acute phase after cerebral ischemia. But MMP-9 may have a different role during delayed phases after stroke. MMP-9 is upregulated in peri-infarct cortex at 7–14 days after stroke and is colocalized with markers of neurovascular remodeling. Treatment with MMP inhibitors at 7 days after stroke suppresses neurovascular remodeling, increases ischemic brain injury and impairs functional recovery at 14 days [74]. MMP-9 also mediate neuroblast cells from the subventricular zone expand and migrate toward damaged tissue during the 2 week recovery period after transient focal cerebral ischemia in mice. And inhibitors of MMPs suppress neurogenic migration from subventricular zone into the striatum [31, 73]. These data suggest that MMP-9 are involved in endogenous mechanisms of neurovascular remodeling in peri-infarct cortex as the brain seeks to heal itself after injury.

## 4. THE REGULATION OF MMP-9 AND THERAPEUTIC IMPLICATION

### 4.1. The Expression and Regulation of MMP-9 

The promoter sequence of MMP-9 contains AP-1 binding site at approximatally -80bp and NF-κB binding site at -600bp [9]. The transcription of MMP-9 is a complex, tight and regulated process [36]. A lot of studies have proved that some inflammatory cytokines and growth factors, such as IL-1β, TNF-α, FGF, EGF can stimulate the secretion of MMP-9 [14, 33, 51, 62]. Inflammatory cells, including monocytes, macrophages and leukocytes are the major originator of MMP-9 in many pathological conditions [4, 12, 25, 61]. In recent years, some researchers focused on the cellular sources of MMP-9 responsible for vascular and parenchymal injury after focal stroke. A research implicated leukocytes, most likely neutrophils, as a key cellular source of MMP-9, which, in turn, promotes leukocyte recruitment, causes BBB breakdown and neuronal injury after transient focal stroke using MMP-9^-/-^ mice and chimeric knockouts lacking either MMP-9 in leukocytes or in resident brain cells [19]. Another study using bone marrow chimeric mice with MMP-9 null and wild-type as donor and recipient demonstrated that bone marrow-derived cells are the major source of MMP-9 in the ischemic brain and BMDC-derived MMP-9 contributes to BBB dysfunction [67]. The inflammatory factors stimulate the secretion of MMP-9 by activating the signalling pathways including MEK1-Erk, P38, PI3K-Akt. At last, these protein kinases regulate the transcription of MMP-9 through activating AP-1 and NF-κB, which subsequently bind to cis elements on the promoter. This promotes the further recruitment of chromatin remodeling complexes, co-activators and general transcriptional machinery to induce MMP-9 expression [36]. At least three classes of co-activators are essential for MMP-9 expression, including CBP/P300, PCAF, CARM1 and GRIP1 [75]. However, not all of the co-activators involved in MMP-9 expression are clear.

MMP-9 are synthesized and secreted into extracellular as inactive zymogen pro-MMP-9. Pro-MMP-9 is activated by disruption of the zinc-thio interaction between the catalytic site and the pro-domain [5]. Plasmin, tPA and uPA are all involved in the process of MMPs activation and plasmin, MMP-2, MMP-3, MMP-13 can activate the pro-MMP-9 directly [9]. In addition, pro-MMP-9 can be activated by NO *via* s-nitrosylation of MMP-9 protein [21, 39]. MMP-9 can be inhibited by all tissue inhibitors of metalloproteinase (TIMP-1, TIMP-2, TIMP-3 and TIMP-4) with a preference of TIMP-1. TIMP-1 can form a complex with pro-MMP-9 interacted with MMP-3 and then dissociated into free pro-MMP-9 and TIMP-1- MMP-3 complex [13]. The process of MMP-9 expression and regulation is summarized in Fig. (**1B**).

### 4.2. Therapeutic Implication

Under the physiological conditions, transcriptional regulation, zymogen activation and endogenous inhibitors could control MMP-9 activity. However, this physiological balance is disturbed after cerebral ischemia. An overexpression of MMP-9 accelerates matrix degradation, disrupts the BBB and relates to hemorrhagic transformation either in animal models or human patients. Since the expression process and the regulation mechanism of MMP-9 have already been known, some therapeutics for cerebral ischemia could be available by regulating the expression and activation of MMP-9. Beside natural MMP inhibitors, treatment with MMP monoclonal antibodies [52], genetic approaches [2] and the broad-spectrum MMP inhibitors such as BB-94 [1, 49], KB-R7785 [29] have been proved to reduce ischemic damage in numerous experimental settings. 

Moreover, many drugs or substances have been proven to attenuate the elevated levels of MMP-9 after ischemia and reduce the damage of cerebral ischemic. 

#### 4.2.1. Tetracycline Derivatives

Minocycline inhibits enzymatic activity of gelatin proteases activated by ischemia after experimental stroke and is likely to be selective for MMP-9 at low doses [37]. Minocycline given to rats twice daily (30mg/kg bodyweight) can reduce infarct sizes, volume and signal intensity of BBB breakdown and improve neuroscore, which was most likely due to inhibition of MMP-2 and MMP-9 [46]. Doxycycline significantly inhibited MMP-9 activity in gel zymography and also suppressed *in situ* gelatinase activity and reduced the laminin degradation and neuronal loss by administration to mice 30 min before and 2 h after ischemia [30]. By inhibiting MMP-2, MMP-9 and plasminogen activators, Doxycycline is shown to decrease injury volumes and protects against damage of the microvessels in focal cerebral ischemia in rats [3]. As described above, tetracycline and tetracycline derivatives have been proved to reduce infarct sizes, protect BBB and have neuroprotective effect in models of cerebral ischemia. They are potential therapeutic agents for acute treatment of cerebral ischemic.

#### 4.2.2. ACE Inhibitors (ACEI) and AT1 Receptor Blockers

Following cerebral ischemia, angiotensin II (venous infusion) increases cerebral edema and mortality by inducing MMP-9 expression in vascular smooth muscle cell through angiotensin type 1 (AT1) receptor and NF-κB pathways [22, 27]. ACEI and AT1 receptor blockers can reduce MMP-9 expression and improve ischemic injury after focal cerebral ischemia. 

Olmesartan, a AT1 receptor blocker can reduce the reactive upregulation in brain angiotensin II, MMP-2, MMP-9 and membrane type 1-MMP in the ischemic area to improve stroke index score, infarct volume, and cerebral edema in cerebral ischemia model. In particular, stroke index score, infarct volume, and cerebral edema were reduced even with a low dose of olmesartan that did not decrease blood pressure [28]. In another study, twelve spontaneously hypertensive stroke-prone rats were randomized into two groups and each group was treated with either an antihypertensive dose of ramipril (an ACE inhibitor) or placebo for 6 months. MMP-9 expression significantly decreased by 37% in the cortex and by 25% in the basal ganglia in animals treated with ramipril [32]. Trandolapril (an ACEI, 5 mg/kg per day) administered orally for 7 days before permanently middle cerebral artery occlusion suppressed MMP-2 and MMP-9 activities at 1 day after MCAO [63]. 

#### 4.2.3. Cyclooxygenase Inhibitors

It has long been recognized that the metabolism of arachidonic acid *via* cyclooxygenase (COX) isozymes is an important contributor to the neuroinflammatory processes following cerebral ischemia. In particular, cyclooxygenase-2 (COX-2) has been shown to be a key player in the evolution of ischemic brain injury [6].

Recent experimental evidences suggest that some non-steroidal anti-inflammatory drugs have therapeutic potential in the treatment of patients with brain ischemia. Indomethacin, an inhibitor of COX-1 and COX-2, significantly reduced the expression and activity of MMP-9 as assessed by immunoblotting and gelatin-substrate zymography, attenuated the brain edema [7]. Treatment with nimesulide markedly attenuates the number of neurons and endothelial cells positive for MMP-2 and MMP-9 [6, 68]. Aspirin, a typical inhibitor of COX, has been proved to inhibit MMP-9 expression [44]. 

Estrogen has shown to suppress COX-2 expression and function induced by interleukin-1β, indicating that estrogen could protect BBB disruption through its inhibition on COX-2 [47]. Estrogens could also attenuate BBB disruption induced by transient cerebral ischemia by inhibition of MMP-2 and MMP-9 activation. This suggests an important role of estrogens as multiple targeting protectants against ischemic stroke on cellular as well as vascular components of central nervous system [34]. 

#### 4.2.4. Antioxidants

Resveratrol, a polyphenolic antioxidant, has neuroprotective activity against cerebral ischemia. The elevated levels of MMP-9 were significantly attenuated in the resveratrol-treated mice as compared to the vehicle MCAO mice suggests that resveratrol has protective effects against acute ischemic stroke which could be attributed to its property against MMP-9 by inhibiting JNK and PKC signal transduction [17, 69]. 

Crocin is the most abundant component with antioxidant effects among the constituents of saffron. Crocin inhibits MMP-9 expression in cortical microvessels in mice with 20 min of bilateral common carotid artery occlusion followed by 24h of reperfusion. Furthermore, it markedly inhibits oxidizing reactions, modulates the ultrastructure of cortical microvascular endothelial cells, inhibits GRK2 translocation from the cytosol to the membrane and reduces ERK1/2 phosphorylation. So, crocin protects the brain against excessive oxidative stress and constitutes a potential therapeutic candidate in transient global cerebral ischemia [76]. 

Quercetin, a natural flavonoid, which is a strong antioxidant and radical scavenger, protects various tissues including neural tissue from ischemia and reperfusion-induced injury by inhibiting MMP-9 activity [10].

#### 4.2.5. Other Drugs and Substances

Some other drugs showed the potential protective effects on cerebral ischemia by downregulating the expression of MMP-9. Calpains and cathepsins are 2 families of cysteine proteases. Recent evidences suggested that calpain/ cathepsin B and MMP-9 are interlinked. E64d, a calpain/cathepsin B inhibitor has been shown to prevent activation of MMP-9 after focal cerebral ischemia and reperfusion injury [64]. 

The post-ischemic MMP-9 overexpression could be regulated by poly (ADP-ribose) polymerase (PARP). The anti-hemorrhagic effect of PJ34, a potent PARP inhibitor was associated with a 57% decrease in MMP-9 overexpression [24]. 

Atorvastatin, an antiatherosclerosis drug, lowers plasma MMP-9 in patients with acute coronary syndrome [20, 65, 70]. Glucocorticoid increases TIMP-1 in the brain endothelial cell line cEND to reduce the levels of MMP-9 [16]. 

## CONCLUSION

MMP-9 provides a new approach for human stroke therapy. The regulations for MMP-9 both on gene level and protein level have been known. So the potential compounds decrease MMP-9 expression by blocking signal pathways or inhibit its activation should be exploited. And knowledge of the three-dimensional (3D) structure of MMPs could provide valuable insights into the structural determinants of selective inhibition of a particular MMP [59]. Further studies about the pathways for MMP-9 expression both in normal and pathological condition are still needed. Moreover, the concrete interaction mechanism between drugs and MMP-9 needs to be further explored. However, MMP-9 has biphasic roles in stroke pathophysiology. It mediates injury during the acute phase and contributes to neurovascular remodeling in the penumbra during the recovery phase. Future investigations should dissect where, when and how MMP-9 increase in damaged brain makes the transition from injury into repair [35]. As more information about MMP-9 we know, anti-MMP-9 therapy for cerebral ischemia could be designed more rationally. 

## Figures and Tables

**Fig. (1):**
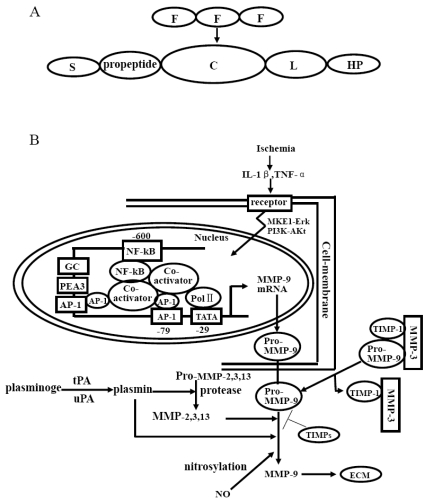
(**A**) The domain structure of MMP-9. S – signal peptide; C – catalytic domain; F – fibronectin type II domain; L – linkage domain; HP – hemopexin like domain. (**B**) Schematic drawing to show the process of MMP-9 expression and regulation. Akt – Protein kinase B (PKB); ECM – extracellular matrix; MMP – matrix metalloproteinase; PI3K – phosphatidylinositol 3-kinase; Pol II – RNA polymerase II; TIMP – tissue inhibitor of metalloproteinase; uPA – urokinase type plasminogen activator; tPA – tissue type plasminogen activator; ERK – extracellular signal activated protein kinase; MEK – mitogen activated ERK activating kinase; NO – nitric oxide. Boxes in nucleus indicate the following cis elements: AP-1 – activator protein-1; PEA3 – polyoma enhancer A binding protein-3; GC – Sp-1 binding site; NF-κB – nuclear factor κB; TATA – TATA box.
